# The effect of fertility treatment and socioeconomic status on neonatal and post-neonatal mortality in the United States

**DOI:** 10.1038/s41372-024-01866-x

**Published:** 2024-01-11

**Authors:** Meesha Sharma, David C. Fineman, Roberta L. Keller, Emin Maltepe, Paolo F. Rinaudo, Martina A. Steurer

**Affiliations:** 1 Department of Pediatrics, University of California, San Francisco Benioff Children’s Hospital, San Francisco, CA USA; 2 Case Western Reserve University PRIME Program, School of Medicine and College of Arts and Sciences, Cleveland, OH USA; 3 Department of Obstetrics, Gynecology and Reproductive Sciences, University of California San Francisco, San Francisco, CA USA; 4 Department of Epidemiology and Biostatistics, University of California San Francisco, San Francisco, CA USA

**Keywords:** Risk factors, Paediatrics, Epidemiology

## Abstract

**Objective:**

To determine the association between fertility treatment, socioeconomic status (SES), and neonatal and post-neonatal mortality.

**Study design:**

Retrospective cohort study of all births (19,350,344) and infant deaths from 2014–2018 in the United States. The exposure was mode of conception—spontaneous vs fertility treatment. The outcome was neonatal (<28d), and post-neonatal (28d–1y) mortality. Multivariable logistic models were stratified by SES.

**Result:**

The fertility treatment group had statistically significantly higher odds of neonatal mortality (high SES OR 1.59; CI [1.5, 1.68], low SES OR 2.11; CI [1.79, 2.48]) and lower odds of post-neonatal mortality (high SES OR 0.87, CI [0.76, 0.996], low SES OR 0.6, CI [0.38, 0.95]). SES significantly modified the effect of ART/NIFT on neonatal and post-neonatal mortality.

**Conclusions:**

Fertility treatment is associated with higher neonatal and lower post-neonatal mortality and SES modifies this effect. Socioeconomic policies and support for vulnerable families may help reduce rates of infant mortality.

## Introduction

The use of assisted reproductive technology (ART), which includes all fertility treatments in which eggs or embryos are handled including in-vitro fertilization (IVF) and intracytoplasmic sperm injection (ICSI), has increased dramatically over the past two decades. In 2018 alone, ART accounted for ~2% (>73,000) of live births [1]; the use of non-IVF fertility treatment (NIFT), which includes the use of ovarian stimulating drugs, accounts for ~3–7% of births [2].

The effect of fertility treatment (ART/NIFT) on infant outcomes remains under study. Adverse neonatal outcomes include increased incidence of preterm birth, low birth weight, and congenital anomalies [2–8]. Adverse infant and childhood outcomes include increased incidence of vascular and cardiac dysfunction [9–14]. However, data on the risk of infant, neonatal and post-neonatal mortality among babies born using ART/NIFT compared to spontaneously conceived babies are conflicting [15–18]. In the US, socioeconomic status (SES) has been shown to be associated with neonatal and post-neonatal outcomes [19, 20] but few studies have explored its effect on infant mortality among babies born through ART/NIFT.

We used a US population-based natality database to investigate infant, neonatal, and post-neonatal mortality following ART/NIFT. We focused on the incidence, timing, and etiology of infant mortality, and the impact of SES on mortality rates. We hypothesized that overall mortality would be increased in ART/NIFT, but mortality differences would be influenced by plurality, prematurity, and maternal socioeconomic status.

## Methods

### Population

We performed a retrospective, population-based cohort study of all live births in the US from 2014–2018. The Center for Disease Control and Prevention (CDC) provides annual cohort linked birth/infant death public use data files (https://www.cdc.gov/nchs/data_access/vitalstatsonline.htm). These datasets contain all live births in the United States linked to death certificates during the first year of life. Detailed documentation of each item collected on birth and death certificates is available at https://www.cdc.gov/nchs/data_access/vitalstatsonline.htm. We evaluated data files starting in 2014, as universal adoption of the revised birth certificate became mandatory for all states at that time.

### Exposure variables

The primary predictor was mode of conception—spontaneous versus use of fertility treatment (ART/NIFT). The revised birth certificate data includes a question about receipt of any fertility treatment. Certificates with a “No” to this question were categorized in the spontaneous conception group. Certificates with a “Yes” answered a follow up question regarding the receipt of ART specifically. Certificates with a “Yes” to this second question were categorized in the ART group. Those with a “Yes” to the first question and “No” to the second question were categorized in the NIFT group. For most of our analyses, the ART and NIFT group were combined as the fertility treatment group. The spontaneous conception group served as the reference category for the analyses. Birth records with missing information about fertility treatment were excluded.

We also assessed the effect of maternal socioeconomic status on our outcome. We defined maternal socio-economic status using education and insurance variables from the birth certificate. Higher socio-economic status (SES) was defined as a maternal education level of community college or university degree, and private insurance. Lower SES was defined as a maternal education of a high school degree or less, and public insurance. Intermediate SES was comprised of all other records. However, our analysis compared only low to high SES.

### Outcomes

The primary outcome was infant mortality (death within the first year of life). We further divided mortality into neonatal (0–28 days) and post-neonatal (29–365 days) mortality. Secondary outcomes were time to death and causes of neonatal and post-neonatal death.

We defined sudden unexpected infant death (SUID) as deaths with the following ICD-10 codes: sudden infant death syndrome (SIDS)—R95, deaths from other ill-defined or unknown causes - R99; and accidental strangulation or suffocation in bed - W75, as per the CDC [21] and prior studies [22].

### Covariates

In addition to SES level, we identified the following covariates a priori as potential confounders in the relationship between mode of conception and infant death: maternal age, self-reported maternal race, body mass index (BMI), cigarette smoking, prematurity, intrauterine growth restriction (IUGR). The social construct of maternal race, as categorized and self-reported in the birth certificate, was included in the analysis as a descriptor of the study population and as a potential confounder, associated with both the exposure and outcome. Non-Hispanic American Indian or Alaskan Native (AIAN), Native Hawaiian or Other Pacific Islander (NHOPI) and those who selected more than one race were categorized together in the “Other” category since the numbers in these categories were small. We adjusted all multivariable models for these covariates. IUGR was defined as birth weight *z*-score less than −1.3 [23, 24], with z-score assigned based on gestational age and sex using the LMS method (lambda for the skew, mu for the median, and sigma for the generalized coefficient of variation) described by Fenton et al. [25, 26].

### Statistical analysis

We used Chi square tests to compare count data and t-tests to compare means of different groups. We used multinomial logit models to assess the trend of births and a logistic model to assess the trend of mortality after fertility treatment.

We used multivariable logistic models to calculate odds ratios (OR) and 95% confidence intervals (95% CI) for infant, neonatal, and post-neonatal mortality among babies born after fertility treatment (ART/NIFT) compared to those born through spontaneous conception, adjusting for the above-mentioned covariates. We stratified our analysis by SES (high versus low) and assessed for interactions between SES and fertility treatment. We constructed survival curves for each conception group by SES and plurality to visually depict timing of death in each group. Finally, using the causes of death data, we identified the most common reasons for neonatal and post-neonatal deaths in each of the conception groups using descriptive statistics.

For supplementary analyses, we assessed ART and NIFT as separate predictors, stratified our results by plurality (single versus multiple gestation), and constructed sequential models, with and without prematurity, to evaluate its effect on infant mortality.

A *p* < 0.05 was considered statistically significant. All analyses were performed using Stata Statistical Software version 17.0 (StataCorp LLC, College Station, TX). No institutional reviewing board approval was necessary since the data used was publicly available and deidentified.

## Results

A total of 19,632,665 live births were documented from 2014–18; 258,963 (1.32%) had missing information regarding mode of conception and were excluded from our study (Supplementary Fig. 1). Of 19,350,344 births, 191,941 (0.99%) followed ART and 118,941 (0.61%) followed NIFT. Compared to spontaneous pregnancies, babies conceived via fertility treatments (ART/NIFT) were more often multiple gestation (29.7% versus 3%), premature (26.45% versus 11.19%), had lower median birth weight z-scores (−0.2 versus −0.12) and had higher rates of congenital malformations (0.42% vs 0.27%) (Table 1). Mothers who received ART/NIFT were more likely to be in the high SES category (73.07% vs. 30.22%).

**Table 1:** Demographic characteristics of births in the United States from 2014–2018.

		Total *N* (%)	Spontaneous Conception *N* (%)	ART/NIFT *N* (%)	*p*-value
Infant Characteristics					
Plurality	Single	18,692,139 (96.6)	18,473,643 (97.03)	218,496 (70.28)	<0.001
	Multiple	658,205 (3.4)	565,819 (2.97)	92,386 (29.72)	
Gestational Age	<37 weeks	2,213,605 (11.44)	2,131,365 (11.19)	82,240 (26.45)	<0.001
	≥37 weeks	17,123,968 (88.49)	16,895,408 (88.74)	228,560 (73.52)	
	Missing	12,771 (0.07)	12,689 (0.07)	82 (0.03)	
Z-score for birth weight	Mean	−0.96	−0.94	−0.19	<0.001
	Median	−0.12	−0.12	−0.2	
	IQR	−0.77, 0.53	−0.77, 0.53	−0.86, 0.46	
Sex	Female	9,450,921 (48.84)	9,299,049 (48.84)	151,872 (48.85)	0.9
Congenital anomalies		52,315 (0.27)	51,002 (0.27)	1313 (0.42)	<0.001
Mode of delivery	Vaginal	13,163,286 (68.03)	13,021,841 (68.39)	141,445 (45.50)	<0.001
	C-section	6,179,588 (31.94)	6,010,216 (31.57)	169,372 (54.48)	
	Unknown/ Missing	7470 (0.04)	7405 (0.04)	65 (0.02)	
NICU admission		1,682,139 (8.69)	1,615,794 (8.49)	66,345 (21.34)	<0.001
Maternal Characteristics					
Age	Teen	1,066,674 (5.51)	1,066,500 (5.6)	174 (0.06)	<0.001
	20-34 years	15,015,152 (77.6)	14,850,747 (78)	164,405 (52.88)	
	≥35 years	3,268,518 (16.89)	3,122,215 (16.4)	146,303 (47.06)	
Race	Non-Hispanic White	10,081,496 (52.1)	9,857,611 (51.77)	223,885 (72.02)	<0.001
	Non-Hispanic Black	2,769,939 (14.31)	2,755,205 (14.47)	14,734 (4.74)	
	Non-Hispanic Asian	1,214,779 (6.28)	1,181,469 (6.21)	33,310 (10.71)	
	Hispanic	4,520,235 (23.36)	4,493,955 (23.60)	26,280 (8.45)	
	Non-Hispanic Other Race (including more than one race)	600,740 (3.1)	595,089 (3.13)	5651 (1.82)	
	Unknown/ Not stated	163,155 (0.84)	156,133 (0.82)	7022 (2.26)	
Insurance	Private	9,382,636 (48.49)	9,103,654 (47.81)	278,982 (89.74)	<0.001
	Medicaid	8,240,991 (42.59)	8,223,501 (43.19)	17,490 (5.63)	
	Self-Pay	820,215 (4.24)	814,669 (4.28)	5546 (1.78)	
	Other	775,951 (4.01)	768,442 (4.04)	7509 (2.42)	
	Missing	130,551 (0.67)	129,196 (0.68)	1355 (0.44)	
SES	High	5,981,771 (30.91)	5,754,602 (30.22)	227,169 (73.07)	<0.001
	Low	7,044,809 (36.41)	7,034,872 (36.95)	9937 (3.2)	
	Other	5,958,767 (30.79)	5,893,391 (30.95)	65,376 (21.03)	
	Missing	364,997 (1.89)	356,597 (1.87)	8400 (2.7)	
Education	High School degree or less	11,475,041 (59.30)	11,415,454 (59.96)	59,587 (19.17)	<0.001
	Higher Degree (Community College/ University degree)	7,634,169 (39.45)	7,389,982 (38.81)	244,187 (78.55)	
	Missing	241,134 (1.25)	234,026 (1.23)	7108 (2.29)	
BMI	Underweight	656,342 (3.39)	649,228 (3.41)	7114 (2.29)	<0.001
	Normal	8,299,572 (42.89)	8,151,798 (42.82)	147,774 (47.53)	
	Overweight	4,898,841 (25.32)	4,821,751 (25.33)	77,090 (24.80)	
	Obese	4,938,400 (25.52)	4,865,696 (25.56)	72,704 (23.39)	
	Missing	557,189 (2.88)	550,989 (2.89)	6200 (1.99)	
Smoker		1,882,965 (9.73)	1,876,608 (9.86)	6357 (2.04)	<0.001
Trimester prenatal care began	No prenatal care	308,670 (1.6)	308,051 (1.62)	619 (0.2)	<0.001
	1st	14,463,318 (74.74)	14,190,013 (74.53)	273,305 (87.91)	
	2nd	3,150,469 (16.28)	3,124,587 (16.41)	25,882 (8.33)	
	3rd	849,057 (4.39)	844,773 (4.44)	4284 (1.38)	
	Missing	578,830 (2.99)	572,038 (3)	6792 (2.18)	
Number of prenatal visits (mean [SD])		13.6 (14.8)	13.6 (14.8)	14.6 (13.5)	<0.001

Birth of infants conceived using ART/NIFT increased from 2014–2018, with almost a 50% increase in ART births (*p* < 0.001) and a 10% increase in NIFT births (*p* < 0.001) (Supplementary Fig. 2a). Neonatal mortality among infants conceived using ART decreased by 32% over the study period (*p* < 0.001) (Supplementary Fig. 2b) while the reduction in neonatal mortality in the NIFT group was not statistically significant (*p* = 0.3). The decrease in post-neonatal mortality among ART/NIFT infants was modest and not significant. Neonatal and post-neonatal mortality among spontaneously conceived infants remained constant over the years (Supplementary Fig. 2b).

**Fig. 1:**
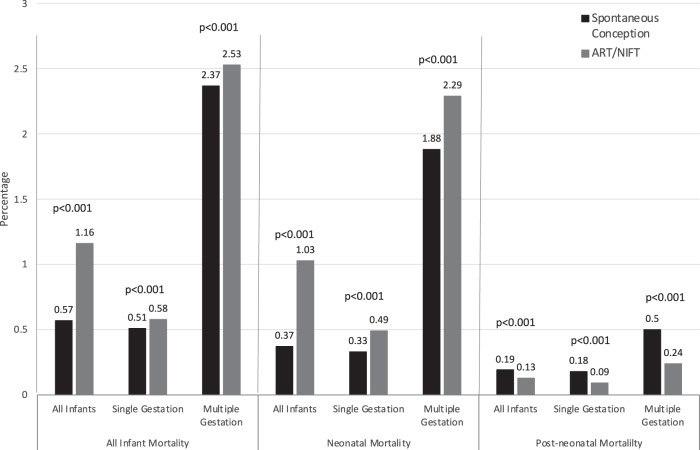
Infant Mortality by Mode of Conception (Spontaneous vs. ART/NIFT) and Plurality (Single vs. Multiple Gestation).

Over the 5-year period, overall infant mortality was 5.8 deaths per 1000 live births (0.58%). It was greater in the ART/NIFT group (1.16%) compared to spontaneously conceived infants (0.57%, *p* < 0.001) (Fig. 1). Neonatal mortality was 3.8 deaths per 1000 live births (0.38%), while post-neonatal mortality was 1.9 deaths per 1,000 live births (0.19%). Compared to the spontaneously conceived group, the ART/NIFT group had a higher neonatal mortality rate (1.03% vs 0.37%) but lower post-neonatal mortality rate (0.13% vs 0.19%) overall, and for singleton and multiple gestation births (Fig. 1, *p* < 0.001 for all comparisons).

After adjusting for confounders, neonates born through ART/NIFT had statistically significantly higher odds of mortality than those who were spontaneously conceived (OR = 1.61, CI 1.54, 1.68) (Table 2). Odds of post-neonatal mortality were lower in the ART/NIFT group (OR = 0.86, CI 0.77, 0.95) and this was statistically significant (Table 2). On stratification, SES was found to be a modifier of the effect of ART/NIFT on infant, neonatal and post-neonatal mortality. Infants in the low SES group had higher odds of neonatal mortality and lower odds of post-neonatal mortality than infants in the high SES group. In a test for interaction, the *p*-value for the interaction term between ART/NIFT and SES was 0.01 for both neonatal and post-neonatal mortality and 0.77 for infant mortality (Table 2). When ART and NIFT were treated as independent predictors, similar trends were seen in mortality outcomes (Supplementary Table 1).

**Table 2:** Crude and adjusted odds ratios of mortality for infants conceived with fertility treatment (ART/NIFT) vs. spontaneous conception stratified by socioeconomic status (SES).

	Overall			High SES			Low SES		
	Infant Mortality	Neonatal Mortality	Post-neonatal Mortality	Infant Mortality	Neonatal Mortality	Post-neonatal Mortality	Infant Mortality	Neonatal Mortality	Post-neonatal Mortality
	OR (CI)	OR (CI)	OR (CI)	OR (CI)	OR (CI)	OR (CI)	OR (CI)	OR (CI)	OR (CI)
**Crude OR**									
**ART/NIFT**^b^	**2.06**	**2.76**	**0.68**	3*	3.59**	1.34***	2.94*	4.39**	0.76***
	**(1.99, 2.13)**	**(2.66, 2.86)**	**(0.62, 0.75)**	**(2.87, 3.14)**	**(3.42, 3.77)**	**(1.18, 1.53)**	**(2.56, 3.37)**	**(3.81, 5.07)**	(0.5, 1.15)
**Adjusted OR**^a^									
**ART/NIFT**^b^	**1.47**	**1.61**	**0.86**	**1.42**	**1.59**	**0.87**	**1.67**	**2.11**	**0.60**
	**(1.42, 1.53)**	**(1.54, 1.68)**	**(0.77, 0.95)**	**(1.35, 1.5)**	**(1.5, 1.68)**	**(0.76, 0.996)**	**(1.43, 1.94)**	**(1.79, 2.48)**	**(0.38, 0.95)**

Bolded values are statistically significant (*p* < 0.05).

^*^p-value for interaction term ART/NIFT * SES = 0.77.

**p-value for interaction term ART/NIFT * SES = 0.01.

***p-value for interaction term ART/NIFT * SES = 0.01.

^a^Models adjusted for maternal age, maternal race, maternal BMI, maternal smoking, plurality (single vs multiple gestation), prenatal care, mode of delivery, prematurity, IUGR (using z-score for birth weight).

^b^Reference group for all models was “Spontaneously conceived infants”.

**Fig. 2:**
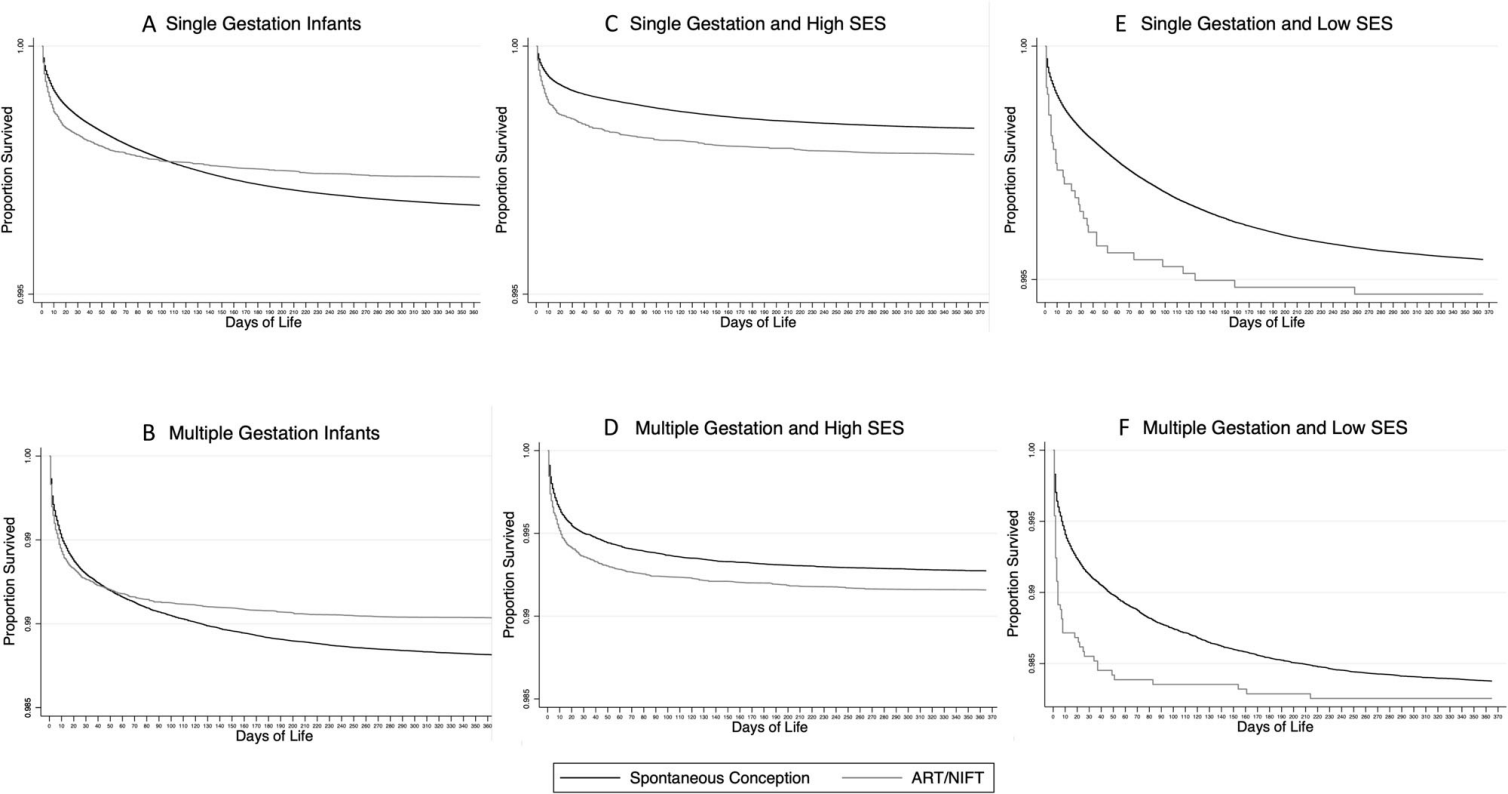
Survival curves by mode of conception stratified by SES. **A**, **C**, **E** Single gestation infants. **B**, **D**, **F** Multiple gestation infants.

Survival curves constructed for the first year of life, including the neonatal period (birth – 28 days) and post-neonatal period (29–365 days), crossed from higher to lower mortality rate following ART/NIFT compared to spontaneous conception at approximately 110–120 days of life for singletons and 50–60 days of life for multiple gestation births (Fig. 2A, B). This crossover effect disappeared after stratification into high and low SES categories, with persistence of lower survival following ART/NIFT in both high and low SES groups (Fig. 2C–F).

**Fig. 3:**
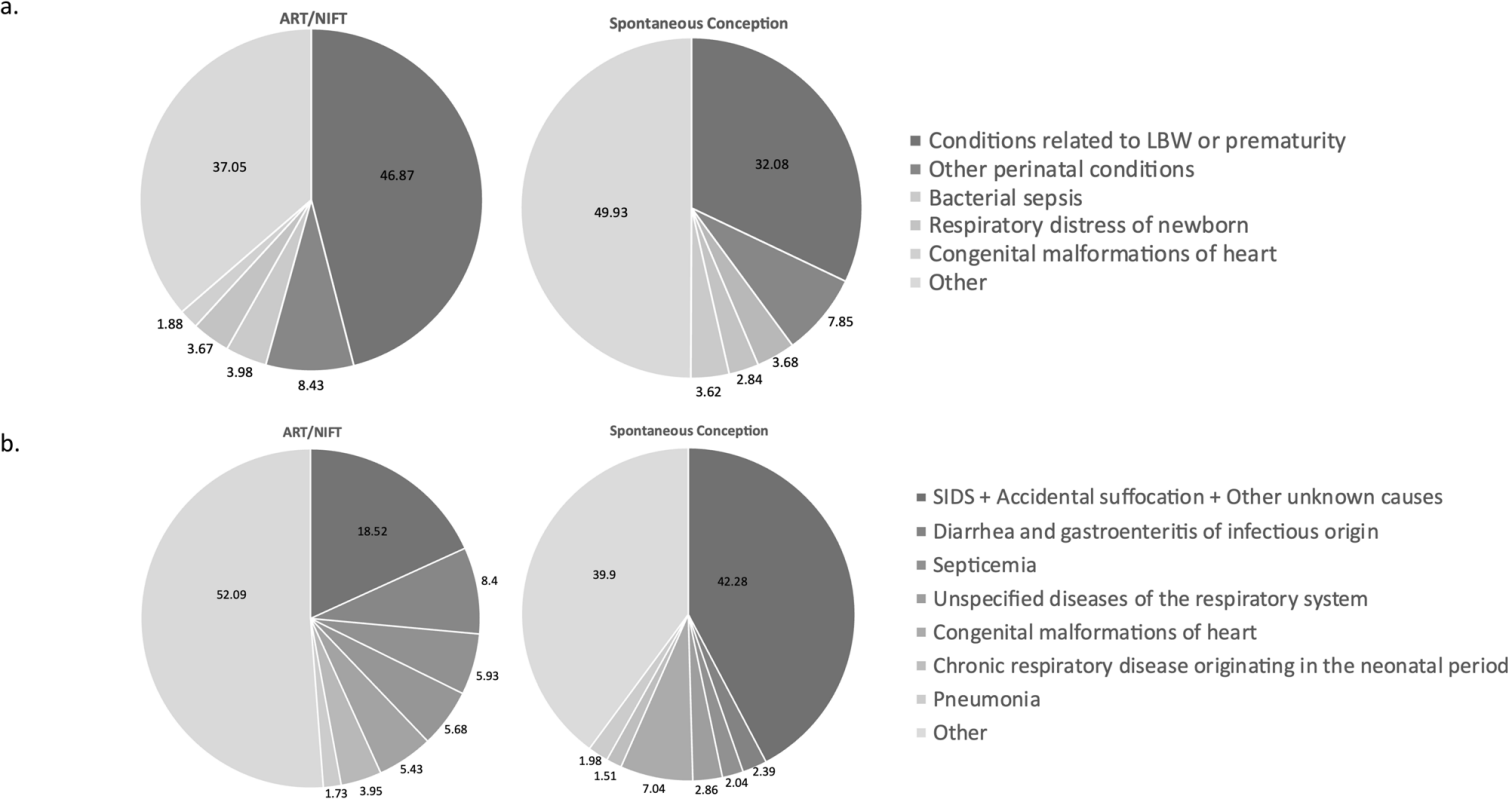
Causes of death. **a** Causes of Neonatal Deaths Among ART/NIFT and Spontaneously Conceived Infants. **b** Causes of Post-Neonatal Deaths Among ART/NIFT and Spontaneously Conceived Infants.

The three most common causes of neonatal death were conditions related to low birth weight or prematurity and other perinatal conditions (including cardiovascular, neurological etc) for both the spontaneously conceived and ART/NIFT groups (Fig. 3a). The single most common cause of post-neonatal death in both groups was SIDS (spontaneous, 17.55% versus ART/NIFT, 9.63%). SUID (including SIDS, other ill-defined or unknown causes and accidental suffocation or strangulation in bed) accounted for 42.28% of post-neonatal deaths among spontaneously conceived children, compared to 18.52% among infants conceived with ART/NIFT (*p* < 0.001) (Fig. 3b).

In supplementary analyses, after adjusting for prematurity, the increased odds of neonatal mortality following ART/NIFT decreased (OR 1.96 [CI 1.88, 2.05] excluding prematurity; OR 1.61 [CI 1.54, 1.68] including prematurity) and the decreased odds of post-neonatal mortality (OR 0.93, CI 0.83, 1.03) further decreased and became statistically significant (OR 0.86, CI 0.77, 0.95) (Supplementary Table 2). However, this confounding effect of prematurity was not seen once data were stratified by plurality. Stratification by plurality revealed an interaction between ART/NIFT and plurality (Supplementary Table 2) for both neonatal and post-neonatal death with higher increased odds of neonatal mortality (multiple gestation OR 1.98, CI 1.87, 2.10; single gestation OR 1.5, CI 1.4, 1.61) and lower decreased odds of post-neonatal mortality (multiple gestation OR 0.92, CI 0.78, 1.07; single gestation OR 0.81, CI 0.7, 0.95) among multiple gestation infants.

## Discussion

We compared the risk of mortality and timing of death among infants conceived with fertility treatment (ART/NIFT) compared to those conceived spontaneously across the United States from 2014–2018. We found that although overall infant mortality following ART/NIFT decreased over the study period, it remained higher than mortality among spontaneously conceived infants, even after adjusting for important confounders. We demonstrated that higher infant mortality following ART/NIFT was driven by higher neonatal mortality, while post-neonatal mortality among ART/NIFT infants was lower. Finally, we demonstrated SES is a significant modifier of the effect of ART/NIFT on neonatal and post-neonatal mortality.

Prior studies of infant mortality following ART/NIFT have demonstrated inconsistent results [4, 6, 27–31]. In a Swedish cohort of singletons, overall infant mortality was higher among those born following ART, but adjusted models did not include gestational age [28]. Post-neonatal mortality was elevated in this cohort, although the effect was not statistically significant [28]. A US population-based study demonstrated higher neonatal mortality among sub-fertile women, but lower infant and neonatal mortality among IVF treated women [4]. The Massachusetts Outcome Study also demonstrated higher perinatal mortality among sub-fertile women compared to fertile women but no difference between fertile and ART treated women [6]. On the other hand, Chang et al found a decreased risk of perinatal death among babies born <28-weeks gestation in a separate US cohort of 3 states, that they attributed to earlier detection and management of adverse conditions of the mother and fetus [29]. Previously, our group demonstrated a two-fold increase in mortality following ART among infants diagnosed with pulmonary vascular disease in a US population-based study, but these differences became insignificant after controlling for gestational age [31]. In a separate single center study, we found a non-significant elevation in 1-year mortality for ART versus spontaneously conceived very preterm infants (<32 weeks) [32].

Although we also found that infant mortality is higher among infants conceived using ART/NIFT, this effect was driven specifically by higher neonatal mortality. The most common causes of neonatal mortality were related to prematurity and low birth weight. Prior studies have demonstrated higher rates of preterm birth, low birth weight and small-for-gestation birthweight in infants born after fertility treatment [4, 5]. Thus, the increased rates of these complications among ART/NIFT groups are likely the reason for higher neonatal mortality in our study population. Biological explanations for increased rates of adverse perinatal outcomes include suboptimal endometrial development and abnormal placentation, which may be precursors of fertility treatment [18]. Prior studies have demonstrated a direct effect of multiple gestation following ART/NIFT on increased rates of prematurity [33]. We found that multiple gestation modifies the effect of ART/NIFT on neonatal mortality. Thus, focus on mitigating these complications including restricting multiple gestation during ART/NIFT should be a focus of on-going efforts to reduce infant mortality, specifically neonatal mortality.

Prior studies have found an association between lower SES and higher rates of neonatal mortality [19, 20, 34–37]. Singh and Kogan demonstrated widening socioeconomic disparities in neonatal mortality in the United States from 1985–2001 [19]. Ratnasiri et al previously demonstrated a “prominent effect” of maternal socio-economic status measured by education, residence in rural areas, insurance and receipt of Supplemental Nutrition Program for Women, Infants and Children (WIC) on infant mortality in a cohort of births in California [34]. In our study, we also found an association between SES and neonatal mortality. The odds of neonatal mortality among ART/NIFT infants were increased in both the low and high SES groups and the increase in odds was greater in the low SES group. Thus, in addition to continued efforts to decrease multiple gestation, preterm birth rates and other complications [38, 39], dedicated socioeconomic resources for those most at risk of disparate neonatal outcomes (those with lower education and government insurance) is paramount to improving neonatal and therefore infant mortality outcomes among those receiving fertility treatment.

Interestingly, odds of post-neonatal mortality were lower in the ART/NIFT group compared to spontaneously conceived infants. In the low SES group, we found a 40% reduction in the odds of post-neonatal mortality, while in the high SES group, odds reduced by 13%. We hypothesize that this difference in the reduction of odds of post-neonatal mortality may be attributed to better access to pre- and post-natal care by mothers in the low SES group receiving fertility treatment compared to those who had spontaneous conception, while access to such care is similar regardless of mode of conception in the high SES group.

Our findings reinforce data from prior studies that have demonstrated an association between SES and post-neonatal mortality [19, 20, 40]. Singh et al showed substantial neighborhood and individual socioeconomic disparities in post-neonatal mortality in the United States [19, 20]. They found that while overall post-neonatal mortality had declined over time, the disparities in post-neonatal mortality had widened with rates declining fastest in the “least socially deprived group”. A similar trend was seen for maternal education level. Other investigators have studied the effect of social policies such as Paid Family Leave on infant outcomes and have found a reduction in post-neonatal mortality rates after implementation of such policies [41], further suggesting post-neonatal mortality is associated more with socioeconomic factors than biological factors. One difference between the above studies and ours is that our study population comprises of

Regarding the etiology of post-neonatal mortality, the most common cause was SIDS regardless of mode of conception. However, SUID comprised 42.28% of post-neonatal deaths in the spontaneously conceived infants but only 18.52% in the ART/NIFT group. Previous studies have suggested an association between SES and SUID. In a systematic review, Spencer and Logan found a significant dose-response association between SUID and measures of socioeconomic status at both the individual and area level, with an increased risk of infant death increasing with greater social adversity [42]. This effect was found to be independent of other variables such as maternal smoking or infant birth weight. In New Zealand, Mitchell et al found a significant association of individual level socioeconomic factors with sudden infant death [43] and Shipstone et al identified that socially vulnerable families had higher number of risk factors for SUDI [44]. Our study also suggests that SES is associated with post-neonatal mortality, but the protective effect is not entirely explained by SES. Further studies are needed to identify other maternal, infant and biological factors [45] that protect ART/NIFT infants from SUID, and therefore post-neonatal mortality.

To our knowledge, this is the largest study of infant outcomes after fertility treatment, and the only one using a nationally representative sample in the US. Despite its strengths, it has limitations. First, we used a database with limited availability of clinical data such as reasons for infertility, previous infertility, fresh vs frozen embryo transfer, or use of donor oocytes. Second, use of fertility treatment is self-reported in the birth certificate and is vulnerable to recall bias and misclassification bias. However, the number of ART/NIFT births from 2014–2018 are similar to those reported by the CDC using data from all fertility clinics in the US for the same years [1, 46–49]. Third, we combined ART and NIFT as the single exposure variable of “fertility treatment” to obtain a sufficient sample size to detect meaningful differences in outcomes. However, in a supplementary analysis, we present our outcomes using 3 separate exposure groups—spontaneous conception, ART and NIFT groups – and trends of the results were consistent. Lastly, we used education and insurance to define SES. However, this definition does not directly account for other crucial factors such as family and social support, or environmental factors such as exposure to second-hand smoke and pollutants that might affect infant outcomes. Such factors may explain some of the residual differences in post-neonatal mortality we found in our study.

In conclusion, infants conceived via fertility treatment from low SES families are at higher risk of neonatal mortality and lower risk of post-neonatal mortality than spontaneously conceived infants. While medical efforts focused on preventing preterm birth and multiple gestation can help improve neonatal outcomes in these infants, socioeconomic policies and support for the most vulnerable families may help reduce infant mortality even further. While further studies are warranted to identify other maternal and infant factors contributing to lower rates of post-neonatal mortality, it remains imperative that further educational and public health efforts are put forth to attenuate the concerning reality of health disparities secondary to socioeconomic differences.

### Supplementary information

Supplementary Tables and Figures Legend

Supplementary Table 1: Crude and Adjusted Odds Ratios (aOR) of Mortality Among Infants Conceived by ART and NIFT Compared to Spontaneous Conception Stratified by SES

Supplementary Table 2: Crude and Adjusted Odds Ratios (aOR) of Mortality Among Infants Conceived with Fertility Treatment (ART/NIFT) Compared to Spontaneous Conception Stratified by Plurality

Supplementary Fig. 1. Flow Diagram of Study Population Inclusion and Exclusion using CDC linked birth and death data files for 2014 – 2018

Supplementary Fig. 2: Birth and Mortality Trends from 2014 - 2018

## Data Availability

We used the public use files provided by the Centers for Disease Control and Prevention (CDC). All data can be accessed via the following link: https://www.cdc.gov/nchs/data_access/vitalstatsonline.htm.

## Code availability

For the STATA code used to analyze the data, please email corresponding author, Meesha Sharma.
