# Supplementary material for: The effect of fertility treatment and socioeconomic status on neonatal and post-neonatal mortality in the United States
Source: J Perinatol. 2024 Jan 11;44(2):187–94. doi: 10.1038/s41372-024-01866-x (PMC10844066; doi:10.1038/s41372-024-01866-x)
Supplement: Supplementary file 1 — Supplementary Tables and Figures Legend [file 41372_2024_1866_MOESM1_ESM.docx]

**SUPPLEMENTARY TABLES LEGEND:**

**Supplementary Table 1:** Crude and Adjusted Odds Ratios (aOR) of Mortality Among Infants Conceived by ART and NIFT Compared to Spontaneous Conception Stratified by SES

**Supplementary Table 2:** Crude and Adjusted Odds Ratios (aOR) of Mortality Among Infants Conceived with Fertility Treatment (ART/NIFT) Compared to Spontaneous Conception Stratified by Plurality

**SUPPLEMENTARY FIGURES LEGEND:**

**Supplementary Figure 1.** Flow Diagram of Study Population Inclusion and Exclusion using CDC linked birth and death data files for 2014 – 2018.

**Supplementary Figure 2**. Birth and Mortality Trends from 2014 – 2018

**Supplementary Figure 2a:** Birth Trends of Infants Conceived with ART and NIFT

**Supplementary Figure 2b:** Mortality Trends Among Infants conceived with ART, NIFT and Spontaneous Conception
