# Supplementary material for: The effect of fertility treatment and socioeconomic status on neonatal and post-neonatal mortality in the United States
Source: J Perinatol. 2024 Jan 11;44(2):187–94. doi: 10.1038/s41372-024-01866-x (PMC10844066; doi:10.1038/s41372-024-01866-x)
Supplement: Supplementary file 2 — Supplementary Table 1: Crude and Adjusted Odds Ratios (aOR) of Mortality Among Infants Conceived by ART and NIFT Compared to Spontaneous Conception Stratified by SES [file 41372_2024_1866_MOESM2_ESM.docx]

**Supplementary Table 1: Crude and Adjusted Odds Ratios (aOR) for Mortality Among Infants Conceived by ART and NIFT Compared to Spontaneous Conception Stratified by SES**

|  | **Overall** | | | **High SES** | | | **Low SES** | | |
| --- | --- | --- | --- | --- | --- | --- | --- | --- | --- |
|  | **Infant Mortality** | **Neonatal Mortality** | **Post-neonatal Mortality** | **Infant Mortality** | **Neonatal Mortality** | **Post-neonatal Mortality** | **Infant Mortality** | **Neonatal Mortality** | **Post-neonatal Mortality** |
|  | **OR (CI)** | **OR (CI)** | **OR (CI)** | **OR (CI)** | **OR (CI)** | **OR (CI)** | **OR (CI)** | **OR (CI)** | **OR (CI)** |
| **Crude OR** | | | | | | | | | |
| **ART** | **2.08** | **2.76** | **0.74** | **3.02** | **3.57** | **1.50** | **2.85** | **4.37** | 0.58 |
|  | **(1.99, 2.17)** | **(2.64, 2.89)** | **(0.65, 0.83)** | **(2.75, 3.18)** | **(3.36, 3.79)** | **(1.29, 1.75)** | **(2.33, 3.48)** | **(3.55, 5.38)** | (0.29, 1.15) |
| **NIFT** | **2.02** | **2.75** | **0.60** | **2.96** | **3.63** | 1.07 | **3.02** | **4.42** | 0.93 |
|  | **(1.92, 2.13)** | **(2.6, 2.91)** | **(0.51, 0.71)** | **(2.86, 3.2)** | **(3.37, 3.92)** | (0.85, 1.35) | **(2.51, 3.64)** | **(3.62, 5.38)** | (0.55, 1.57) |
| **Adjusted OR^+^** | | | | | | | | | |
| **ART** | **1.44** | **1.54** | 0.93 | **1.39** | **1.53** | 0.94 | **1.44** | **1.79** | 0.53 |
|  | **(1.37, 1.51)** | **(1.46, 1.62)** | (0.82, 1.06) | **(1.31, 1.48)** | **(1.43, 1.64)** | (0.80, 1.11) | **(1.15, 1.82)** | **(1.40, 2.29)** | (0.27, 1.06) |
| **NIFT** | **1.53** | **1.73** | **0.74** | **1.47** | **1.69** | **0.73** | **1.89** | **2.43** | 0.67 |
|  | **(1.44, 1.63)** | **(1.62, 1.84)** | **(0.62, 0.88)** | **(1.36, 1.60)** | **(1.55, 1.84)** | **(0.58, 0.93)** | **(1.54, 2.31)** | **(1.96, 3.02)** | (0.37, 1.21) |

**^+^Models adjusted for maternal age, maternal race, maternal BMI, maternal smoking, plurality (single vs multiple gestation), prenatal care, mode of delivery, prematurity, IUGR (using z-score for birth weight)**

**^++^Reference group for all models was “Spontaneously conceived infants”**

**^+++^Bolded values are statistically significant (p<0.05)**
