# Supplementary material for: The effect of fertility treatment and socioeconomic status on neonatal and post-neonatal mortality in the United States
Source: J Perinatol. 2024 Jan 11;44(2):187–94. doi: 10.1038/s41372-024-01866-x (PMC10844066; doi:10.1038/s41372-024-01866-x)
Supplement: Supplementary file 3 — Supplementary Table 2: Crude and Adjusted Odds Ratios (aOR) of Mortality Among Infants Conceived with Fertility Treatment (ART/NIFT) Compared to Spontaneous Conception Stratified by Plurality [file 41372_2024_1866_MOESM3_ESM.docx]

**Supplementary Table 2: Crude and Adjusted Odds Ratios (aOR) of Mortality Among Infants Conceived with Fertility Treatment (ART/NIFT) Compared to Spontaneous Conception Stratified by Plurality**

|  | **Overall** | | | **Single gestation** | | | **Multiple gestation** | | |
| --- | --- | --- | --- | --- | --- | --- | --- | --- | --- |
|  | **Infant Mortality** | **Neonatal Mortality** | **Post-neonatal Mortality** | **Infant Mortality** | **Neonatal Mortality** | **Post-neonatal Mortality** | **Infant Mortality** | **Neonatal Mortality** | **Post-neonatal Mortality** |
|  | OR (CI) | OR (CI) | OR (CI) | OR (CI) | OR (CI) | OR (CI) | OR (CI) | OR (CI) | OR (CI) |
| **Crude OR** | | | | | | | | | |
| **ART/NIFT** | **2.06*** | **2.76**** | **0.68***** | **1.13** | **1.5** | **0.48** | **1.07** | **1.23** | **0.48** |
|  | **(1.99, 2.13)** | **(2.66, 2.86)** | **(0.62, 0.75)** | **(1.07, 1.20)** | **(1.41, 1.59)** | **(0.41, 0.55)** | **(1.02, 1.12)** | **(1.17, 1.29)** | **(0.42, 0.55)** |
| **Model adjusted for all covariates excluding prematurity^+^** | | | | | | | | | |
| **ART/NIFT** | **1.73** | **1.96** | 0.93 | **1.3** | **1.44** | **0.81** | **1.72** | **1.94** | 0.9 |
|  | **(1.66, 1.80)** | **(1.88, 2.05)** | (0.83, 1.03) | **(1.22, 1.39)** | **(1.35, 1.55)** | **(0.70, 0.94)** | **(1.63, 1.82)** | **(1.83, 2.06)** | (0.77, 1.05) |
| **Adjusted OR^+^** | | | | | | | | | |
| **ART/NIFT** | **1.47** | **1.61** | **0.86** | **1.33** | **1.5** | **0.81** | **1.76** | **1.98** | 0.91 |
|  | **(1.42, 1.53)** | **(1.54, 1.68)** | **(0.77, 0.95)** | **(1.25, 1.42)** | **(1.40, 1.61)** | **(0.70,0.95)** | **(1.66, 1.86)** | **(1.87, 2.10)** | (0.78, 1.07) |

***p-value for interaction term ART/NIFT * plurality = 0.128**

****p-value for interaction term ART/NIFT * plurality = 0.000**

*****p-value for interaction term ART/NIFT * plurality = 0.982**

^+^Models adjusted for maternal age, maternal race, maternal BMI, maternal smoking, plurality (single vs multiple gestation), prenatal care, mode of delivery, prematurity, IUGR (using z-score for birth weight)

^++^Reference group for all models was “Spontaneously conceived infants”

^+++^Bolded values are statistically significant (p<0.05)
