# Supplementary figures and images for: The effect of fertility treatment and socioeconomic status on neonatal and post-neonatal mortality in the United States
Source: J Perinatol. 2024 Jan 11;44(2):187–94. doi: 10.1038/s41372-024-01866-x (PMC10844066; doi:10.1038/s41372-024-01866-x)

Supplementary Figure 1: Flow Diagram of Study Population Inclusion and Exclusion

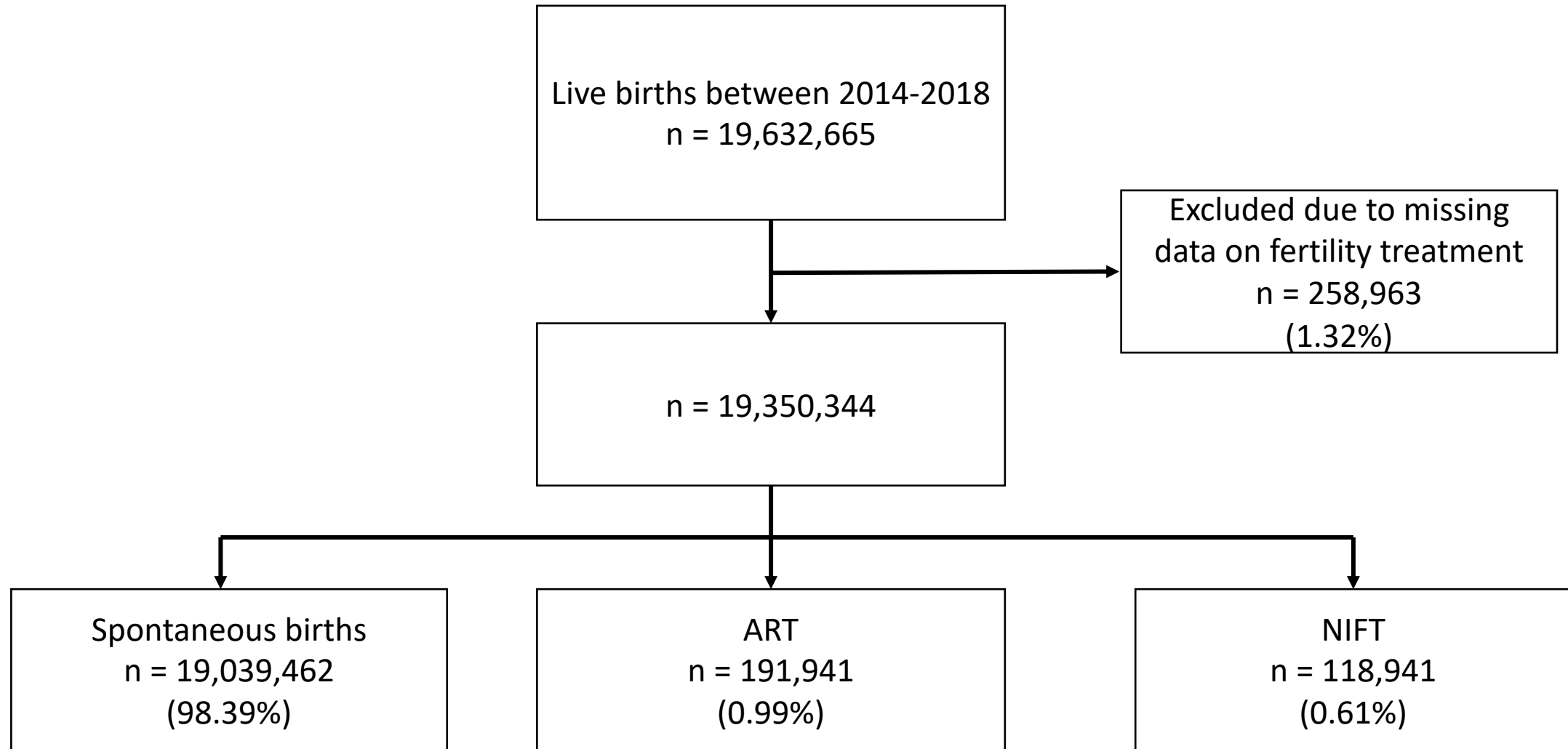

Supplement: Supplementary file 4 — Supplementary Fig. 1. Flow Diagram of Study Population Inclusion and Exclusion using CDC linked birth and death data files for 2014 – 2018 [file 41372_2024_1866_MOESM4_ESM.pdf]
