# Supplementary material for: The effect of fertility treatment and socioeconomic status on neonatal and post-neonatal mortality in the United States
Source: J Perinatol. 2024 Jan 11;44(2):187–94. doi: 10.1038/s41372-024-01866-x (PMC10844066; doi:10.1038/s41372-024-01866-x)

Supplementary Figure 2: Birth and Mortality Trends from 2014 - 2018

Supplementary Figure 2a: Birth Trends

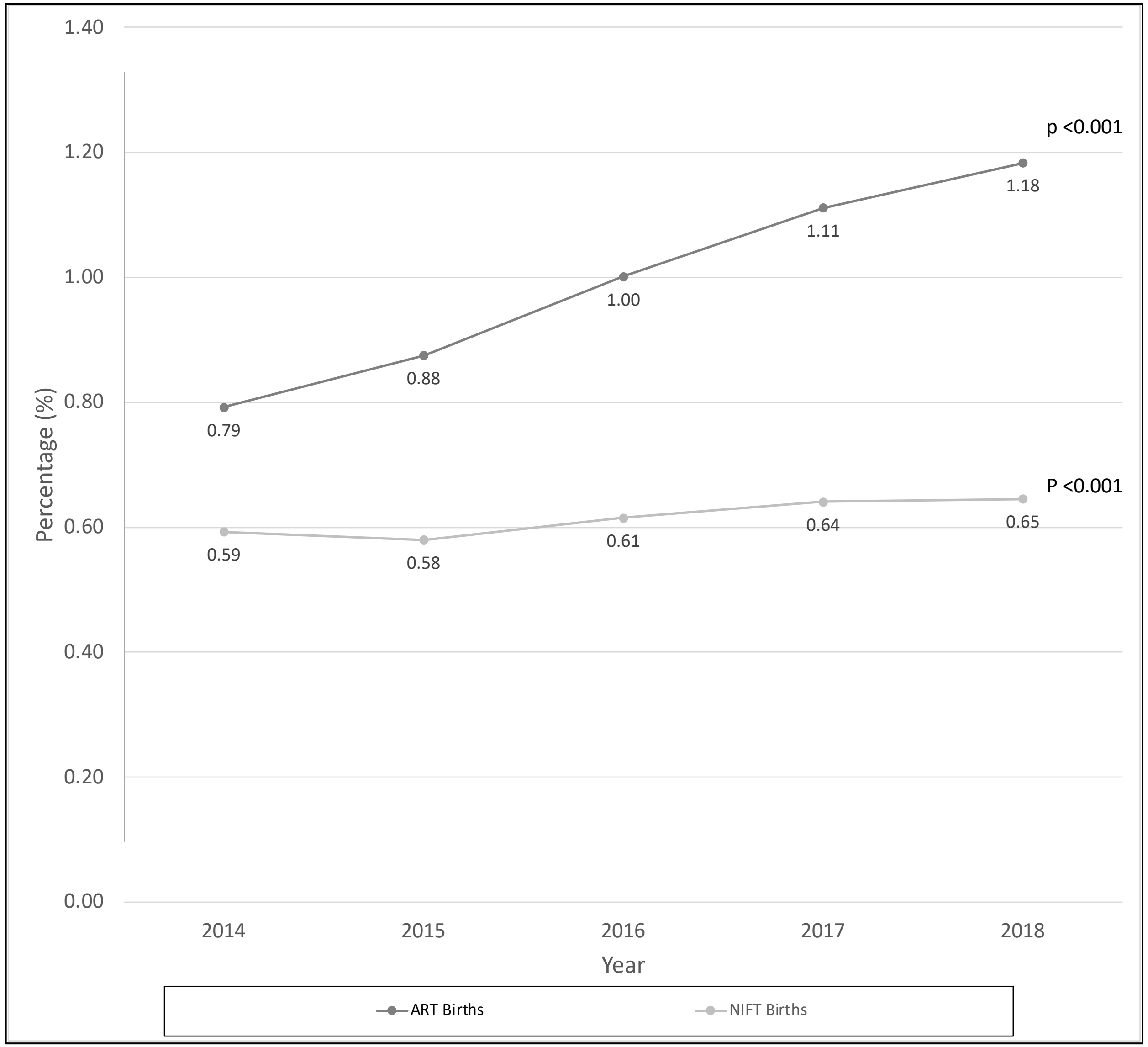

Supplementary Figure 2b: Mortality Trends

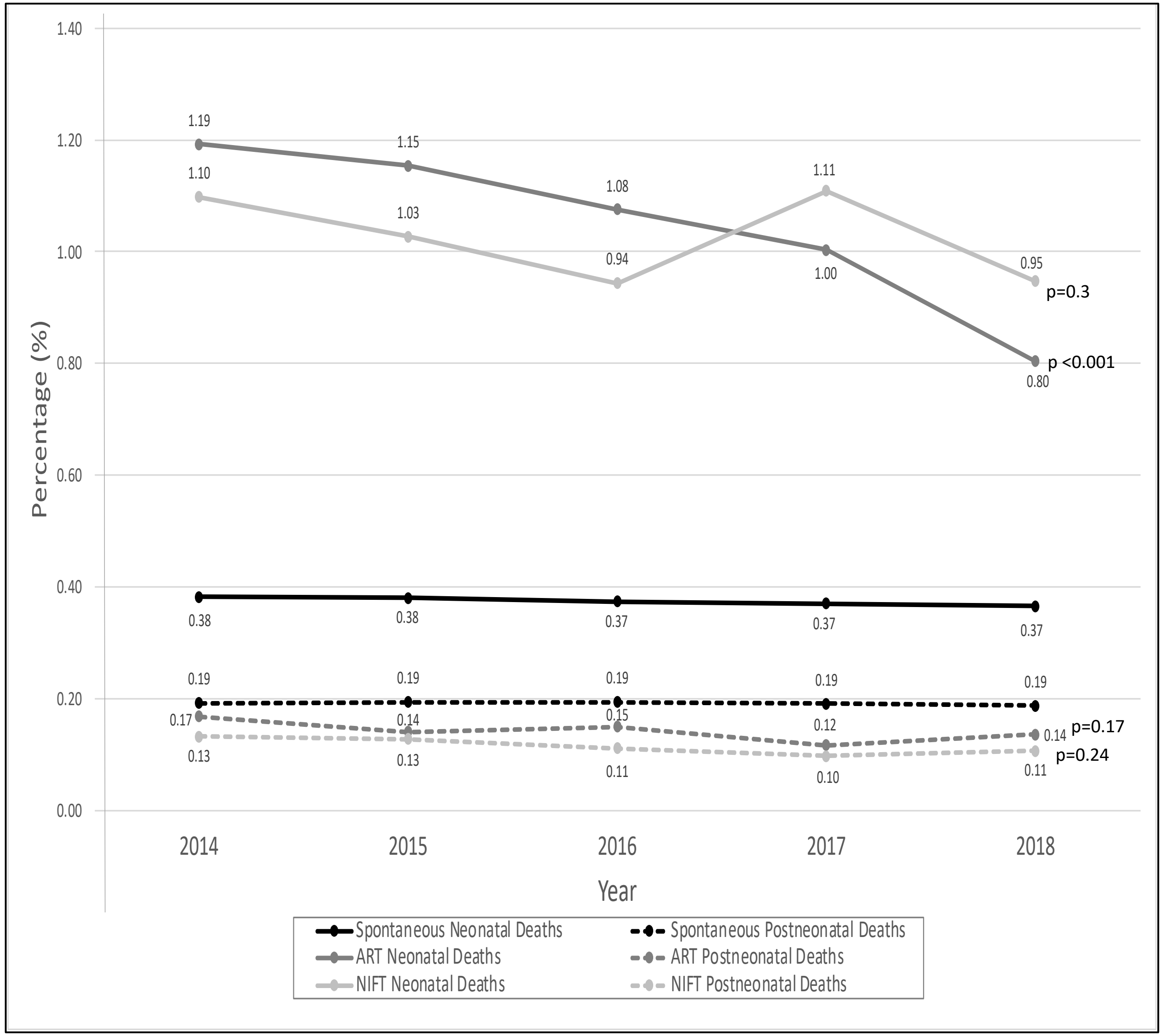

Supplement: Supplementary file 5 — Supplementary Fig. 2: Birth and Mortality Trends from 2014 - 2018 [file 41372_2024_1866_MOESM5_ESM.pdf]
